# The Evolution of Hand Injuries at a State’s Only Level I Trauma Center: A Look From the 1980s Through the Global Pandemic

**DOI:** 10.7759/cureus.54882

**Published:** 2024-02-25

**Authors:** Muntazim Mukit, Martin G McCandless, John C Davidson, Samuel Hopper, Jacob I Jabbour, Stephen F Davidson, Marc Walker

**Affiliations:** 1 Surgery, University of Mississippi Medical Center, Jackson, USA

**Keywords:** covid-19, hand surgeon, acute trauma care, upper extremity trauma, hand trauma management, practice patterns, hand trauma, epidemiology

## Abstract

Background: The purpose of this study is to evaluate any changes to hand trauma in the past three decades and through the COVID-19 pandemic. We hypothesized that improved consumer safety regulations, changes in access to care, and the impact of a global pandemic, among other variables, have significantly influenced the mechanisms and treatment of hand injuries between the 1980s, 2010s (pre-COVID-19), and 2020s (post-COVID-19).

Methods: A retrospective single-center review was performed at the only level I trauma center in Mississippi, identifying all hand trauma consultations between 2012-2019 and 2020-2021, compared to aggregated data from 1989.

Results: Car accidents, gunshots, saw injuries, door injuries, and falls increased in 2012-2019 and 2020-2021 compared to 1989, whereas knife injuries, glass injuries, industrial injuries, and burns decreased. Crush injuries, de-gloving injuries, and lacerations with irregular edges were increased in recent cohorts, corresponding with increased amputations and tissue loss. Skin and subcutaneous injuries decreased in modern cohorts, corresponding with a decreased ability for primary skin repair and the need for more flaps. Additionally, while hospitalizations have increased, patients have improved follow-up.

Conclusions: The nature of hand trauma has changed significantly over the past three decades. Increased numbers of cars and greater access to firearms might have led to increased rates of high-energy trauma, whereas burn and industrial injuries have decreased, potentially secondary to improved safety efforts. Despite increased overall hand trauma, time to treatment and follow-up have improved. Through this study, we can be more cognizant of the evolution of hand trauma in the modern era. This can allow improved access to care and further refine management to optimize functionality for hand injuries.

## Introduction

Hand injuries have been a part of society since antiquity. It has been reported that Hippocrates discussed the reduction and stabilization of wrist, hand, and finger fractures in Ancient Greece, while Galen described hand infections as early as 166 CE [1]. The nature of hand trauma has undoubtedly changed over time with the advent of technology and industrialization. Notably, based on available studies, the mechanism of hand trauma in the 1980s can be postulated to be different from what we see today. This collective hypothesis stems from the epidemiology studies on hand trauma from Sweden, the Netherlands, Denmark, Norway, Poland, the United Arab Emirates, and India [2-7], as well as other studies that reviewed hand trauma examined in specific time periods [8-14] and limited populations such as children in emergency department settings [15-16]. Nearly all currently available studies have looked into hand trauma during a consecutive, limited time period [3-16]; however, there is one study from Sweden characterizing hand trauma in a non-consecutive time period [2].

The purpose of our study was to determine whether hand trauma has evolved in the past three decades and during the COVID-19 pandemic. Information was collected regarding all patients suffering from hand trauma at the only level I trauma center in the state of Mississippi during three distinct time periods: 1989, 2012-2019, and 2020-2021.

We hypothesize that improved consumer safety regulations, changes in access to care, and the impact of a global pandemic, amongst other variables, have significantly influenced the mechanisms and treatment of hand injuries between the 1980s, 2010s (pre-COVID-19), and 2020s (post-COVID-19).

This article was previously presented at the 2022 SESPRS Resident Glancy Competition on June 12, 2022, as a podium presentation, at the 2022 ASSH Annual Meeting on October 1, 2022, as an e-poster, and at the annual 2022 PSTM on October 28, 2022, as a podium presentation.

## Materials and methods

This study was conducted in a two-stage process. First, a prospective chart review was done in 1989 for all patients suffering from hand trauma at the University of Mississippi Medical Center (UMMC). The paper charts were reviewed for every presenting hand trauma consultation. The data was collected over the course of one year and then compiled in an aggregate manner. Data variables such as age, sex, cause of injury, type of injury, tissue injury, bony injury, treatment, presence of tissue loss, number of operations, percentage of patients hospitalized, number of days hospitalized, workman’s compensation cases, time to maximum recovery (when the patient was either discharged from clinic or when they were last seen), complications (such as infection or tissue necrosis), need for follow-up, completion of follow-up, injury to treatment time >12 hours, and being admitted for a hand problem were all collected.

In the second stage, a retrospective chart review was conducted, reviewing all patients at the same institution suffering from hand trauma between 2012 and 2021. The electronic medical record was queried for all patients who received a consult order for pediatric plastic surgery, plastic surgery, hand surgery, or orthopedic surgery involving any hand, digit, finger, or wrist problem. All consults were immediately seen by a plastic surgery resident, orthopedic surgery resident, or plastic surgery hand fellow, and then staffed with an attending. Every patient was seen by the attending in the hospital setting, if admitted, or in the outpatient subspecialty clinic.

A total of 1538 charts were retrieved using the search criteria. After excluding duplicate charts or hand consultations that were not trauma-related, there were a total of 920 charts. To analyze the effect of the global COVID-19 pandemic on hand trauma, the 2012-2021 cohort was divided into two groups: pre-COVID-19 (2012-2019) and post-COVID-19 (2020-2021). All variables that were collected in 1989 were collected for the 2012-2021 group. Both inpatient and outpatient data were reviewed through the electronic medical record. Institutional review board approval was obtained from the University of Mississippi Medical Center Institutional Review Board (approval number: 2021-1123).

Data analysis was done using the chi-square test for categorical variables, Fisher’s exact test for categorical variables with small absolute numbers per category, and an independent t-test for continuous variables. Significance was set at a p-value of less than 0.05.

## Results

A total of 979, 713, and 207 patients were in the 1989, 2012-2019, and 2020-2021 cohorts, respectively. The comprehensive table listing absolute numbers and percentages for each cohort is listed in Table 1.

**Table 1:** Differences in hand trauma between the years 1989, 2012-2019 and 2020-2021 N/A: not applicable, MVA: motor vehicle accident, GSW: gunshot wound, TBSA: total body surface area, ATV: all-terrain vehicle, STSG: split thickness skin graft, FTSG: full-thickness skin graft, I&D: irrigation and debridement *Fisher's exact test performed, **Two-sided independent T-test performed

	Year/cohort			
	1989 (N=979)	2012-2019 (N=713)	2020-2021 (N=207)	p-value
Age (years)	All ages (range unknown)	1-93	1-92	
Sex (male/female)	627 (64%)/352 (36%)	543 (76%)/170 (24%)	140 (68%)/67 (32 %)	<0.001
Cause of injury				
MVA	78 (8 %)	108 (15%)	47 (23%)	<0.001
GSW	56 (5.7 %)	77 (11%)	29 (14%)	<0.001
Knife	264 (27 %)	51 (7.1%)	11 (5%)	<0.001
Saw	29 (3 %)	50 (7.0%)	11 (5%)	<0.001
Snake bite	3 (0.3 %)	2 (0.28%)	0%	0.732
Dog bite	28 (2.9 %)	15 (2.1%)	2 (1%)	0.223
Human bite	24 (2.5 %)	7 (1.0%)	1 (0.5%)	0.025
Cat bite	4 (0.4 %)	1 (0.14%)	0 %	0.418
Industrial	182 (18.6 %)	78 (10.9%)	12 (6%)	<0.001
1^st^ degree burn	10 (1.0 %)	0 (0%)	1 (0.5%)	0.023
2^nd^ degree burn <1% TBSA	52 (5.3 %)	4 (0.60%)	0%	<0.001
2^nd^ degree burn >1% TBSA	25 (2.6 %)	0 (0%)	0%	<0.001
3^rd^ degree burn	8 (0.8 %)	2 (0.3%)	1 (0.5%)	0.350
Glass	136 (13.9 %)	43 (6.0%)	8 (4%)	<0.001
Door	27 (2.8 %)	75 (10.5%)	16 (8%)	<0.001
Fall	53 (5.4 %)	70 (9.8%)	29 (14%)	<0.001
Fishing	N/A	0 (0%)	1 (0.5%)	0.255*
Blast injury	N/A	5 (0.7%)	0%	0.593*
Punching	N/A	13 (1.8%)	6 (3%)	0.402*
Crush	N/A	17 (2.3%)	9 (4%)	0.133
Assault	N/A	16 (2.2%)	5 (2%)	0.797*
Fan	N/A	4 (0.6%)	1 (0.5%)	>0.99*
Firework	N/A	0 (0%)	1 (0.5%)	0.255*
Arrow	N/A	0 (0%)	1 (0.5%)	0.255*
Fish hook	N/A	1 (0.14%)	1 (0.5%)	0.400*
Fish barb	N/A	1 (0.14%)	N/A	N/A
Nail gun	N/A	6 (0.84%)	1 (0.5%)	>0.99*
Unknown	N/A	0 (0%)	1 (0.5%)	0.255*
ATV	N/A	7 (1.0%)	3 (1.5%)	0.702*
Toy oven	N/A	0 (0%)	1 (0.5%)	0.255*
Lawnmower	N/A	6 (0.84%)	1 (0.5%)	>0.99*
Sports	N/A	20 (2.8%)	2 (1%)	0.194*
Boat propeller/boating	N/A	2 (0.28%)	N/A	N/A
Ring	N/A	1 (0.14%)	N/A	N/A
Splinter	N/A	1 (0.14%)	N/A	N/A
Bike	N/A	2 (0.28%)	N/A	N/A
Lamp	N/A	2 (0.28%)	N/A	N/A
Iatrogenic	N/A	1 (0.14%)	N/A	N/A
Go cart	N/A	1 (0.14%)	N/A	N/A
Changing tire	N/A	1 (0.14%)	N/A	N/A
Hedge trimmer	N/A	1 (0.14%)	N/A	N/A
Fence	N/A	1 (0.14%)	N/A	N/A
Metal can	N/A	2 (0.28%)	N/A	N/A
Slip injury	N/A	1 (0.14%)	N/A	N/A
Opening door knob	N/A	1 (0.14%)	N/A	N/A
Folding chair injury	N/A	1 (0.14%)	N/A	N/A
Rope	N/A	1 (0.14%)	N/A	N/A
Puncture	N/A	1 (0.14%)	N/A	N/A
Type of injury				
Abrasion	41 (4.2 %)	79 (11%)	9 (4%)	<0.001
Clean cut	465 (47.5 %)	83 (11.6%)	7 (3%)	<0.001
Laceration irregular edges	295 (30.1 %)	324 (45%)	100 (48%)	<0.001
Crush	76 (7.8 %)	166 (23%)	78 (38%)	<0.001
Degloved	7 (0.7 %)	45 (6.3%)	7 (3%)	<0.001
Hyperextension	N/A	0 (0%)	1 (0.5%)	0.255*
Burn	N/A	0 (0%)	1 (0.5%)	0.255*
Puncture	N/A	17 (2.3%)	2 (1%)	0.274*
Tissue injury				
Skin	959 (98 %)	528 (74%)	139 (67%)	<0.001
Muscle	118 (12.1 %)	61 (8.5%)	32 (15%)	0.008
Nerve	89 (9.1 %)	56 (7.9%)	17 (8%)	0.657
Subcutaneous tissue	698 (71.3 %)	408 (57%)	100 (48%)	<0.001
Flexor tendon	77 (7.9 %)	52 (7.2%)	20 (10%)	0.536
Extensor tendon	64 (6.5 %)	72 (10%)	19 (9%)	0.026
Joint capsule	43 (4.4 %)	17 (2.3%)	10 (5)	0.063
Tendon sheath	41 (4.2 %)	23 (3.2%)	17 (8%)	0.007
Artery	36 (3.7 %)	25 (3.5%)	8 (4%)	0.966
Amputation	29 (3.0 %)	45 (6.3%)	12 (6%)	0.003
Nail bed	N/A	69 (9.7%)	28 (14%)	0.112
Nail plate avulsion	N/A	1 (0.14%)	0%	>0.99*
Volar plate	N/A	1 (0.14%)	0%	>0.99*
Collateral ligament	N/A	3 (0.42%)	0%	>0.99*
Bony injury				
Simple fracture	26 (2.7 %)	188 (26%)	37 (18%)	<0.001
Comminuted fracture	37 (3.8 %)	169 (24%)	77 (37%)	<0.001
Open fracture	64 (6.5 %)	149 (21%)	20 (10%)	<0.001
Bone loss	18 (1.8 %)	31 (4.3%)	2 (1%)	0.002
Dislocation	N/A	12 (1.7%)	5 (2%)	0.556*
Treatment				
Primary skin repair	564 (57.6 %)	419 (59%)	85 (41%)	<0.001
STSG needed	20 (2.0 %)	10 (1.4%)	3 (1%)	0.576
FTSG	N/A	8 (1.1%)	1 (0.5%)	0.692*
Flap needed	17 (1.7 %)	6 (0.84%)	13 (6%)	<0.001
Vasculature repair	37 (3.8 %)	8 (1.1%)	5 (2%)	0.003
Primary repair, vein	3 (0.3 %)	0 (0%)	0 %	0.244
Vein graft, vein	0 (0 %)	0 (0%)	0 %	>0.99
Primary repair, artery	21 (2.1 %)	7 (1.0%)	3 (1.4%)	0.172
Vein graft, artery	1 (0.1 %)	1 (0.14%)	2 (1%)	0.042
Bone, stable	26 (2.7 %)	166 (23%)	69 (33%)	<0.001
External fixation	44 (4.5 %)	72 (10%)	8 (4%)	<0.001
Internal fixation emergent	40 (4.1 %)	7 (1.0%)	1 (0.5%)	<0.001
Internal fixation delayed	17 (1.7 %)	101 (14%)	35 (17%)	<0.001
Bone graft	20 (2.0 %)	4 (0.6%)	1 (0.5%)	0.016
Nerve repair, primary	49 (5.0 %)	22 (3.0%)	10 (5%)	0.142
Nerve repair, graft	3 (0.3 %)	10 (1.4%)	0 %	0.012
Amputation	N/A	32 (4.4%)	12 (6%)	0.437
Ray amputation	N/A	1 (0.14%)	N/A	N/A
Tendon repair	N/A	64 (9.0%)	20 (10%)	0.763
Tendon repair w/ graft	N/A	2 (0.28%)	0%	>0.99*
Skin substitute	N/A	2 (0.29%)	2 (1%)	0.220*
Wound vac	N/A	0 (0%)	2 (1%)	0.050*
I&D	N/A	11 (1.5%)	N/A	N/A
Foreign body removal	N/A	3 (0.42%)	2 (1%)	0.314*
Fasciotomy	N/A	2 (0.28%)	2 (1%)	0.220*
Carpal tunnel release	N/A	1 (0.14%)	1 (0.5%)	0.400*
Local wound care	N/A	16 (2.2%)	6 (3%)	0.606*
Joint fixation	N/A	1 (0.14%)	1 (0.5%)	0.400*
Capsulorraphy	N/A	2 (0.28%)	N/A	N/A
Nail bed repair	N/A	55 (7.7%)	18 (8.7 %)	0.645
Repair collateral ligament	N/A	2 (0.28%)	1 (0.5%)	0.535*
Repair volar plate	N/A	1 (0.14%)	1 (0.5%)	0.400*
Tendon transfer	N/A	0 (0%)	1 (0.5%)	0.255*
Reattachment	N/A	5 (0.70%)	0%	0.593*
Open reduction (dislocation)	N/A	2 (0.28%)	N/A	N/A
Closed reduction (dislocation)	N/A	2 (0.28%)	N/A	N/A
Neurolysis	N/A	2 (0.28%)	N/A	N/A
Complications				
Infection	80 (8.2 %)	16 (2.2%)	3 (1.5%)	0.590*
Necrosis	8 (0.8 %)	16 (2.2%)	2 (1%)	0.391*
Delayed healing	N/A	1 (0.14%)	2 (1%)	0.129*
Amputation	N/A	6 (0.84%)	2 (1%)	>0.99*
Stiffness	N/A	16 (2.2%)	2 (1%)	0.391*
Flap ischemia	N/A	0 (0%)	1 (0.5%)	0.255*
Ulnar nerve motor weakness	N/A	0 (0%)	1 (0.5%)	0.255*
Keloid	N/A	0 (0%)	1 (0.5%)	0.255*
Neuroma	N/A	3 (0.42%)	1 (0.5%)	>0.99*
Tendon rupture	N/A	1 (0.14%)	0%	>0.99*
Pain	N/A	4 (0.56%)	0%	0.580*
Malunion	N/A	1 (0.14%)	0%	>0.99*
Non union	N/A	1 (0.14%)	0%	>0.99*
Dehiscence	N/A	3 (0.42%)	0%	>0.99*
Joint contracture	N/A	5 (0.70%)	0%	0.593*
External neurolysis	N/A	1 (0.14%)	0%	>0.99*
Hook nail deformity	N/A	1 (0.14%)	0%	>0.99*
Graft loss	N/A	1 (0.14%)	0%	>0.99*
Hematoma	N/A	1 (0.14%)	0%	>0.99*
Hardware failure	N/A	1 (0.14%)	0%	>0.99*
Rotational deformity	N/A	1 (0.14%)	0%	>0.99*
Hypersensitivity	N/A	1 (0.14%)	0%	>0.99*
Miscellaneous				
Tissue loss	50 (5.1 %)	186 (26%)	94 (45%)	<0.001
Number of operations	Not recorded	0.42 (avg)	0.57 (avg)	0.116**
Hospitalized	203 (20.8 %)	198 (28%)	68 (33%)	<0.001
Number of days	Not recorded	6 (avg)	9 (avg)	0.057**
Workman’s composition	26 (2.7 %)	7 (1.0%)	3 (1.5%)	0.039
Time to max recovery (days)	Not recorded	87 (avg)	62 (avg)	0.042**
Transferred care	Not recorded	3 (0.42%)	2 (1.0%)	0.314*
Lost to follow-up/missing data	N/A	297 (42%)	52 (25%)	<0.001
Follow-up needed	798 (81.5 %)	684 (96%)	198 (96%)	<0.001
Follow-up done	519 (53 %)	428 (60%)	156 (79%)	<0.001
Injury to treatment time >12 hours	92 (9.4 %)	172 (24%)	5 (2%)	<0.001
Admitted for hand problem	24 (2.5 %)	97 (14%)	11 (5%)	<0.001

The exact age range of patients in the 1989 cohort is unknown, but per the senior author who collected the data, all ages were treated. Meanwhile, the age ranges in the 2012-2019 and 2020-2021 cohorts were 1-93 and 1-92, respectively. The composition of male/female patients differed between the cohorts (64% (627) male/36% (352) female in 1989 vs. 76% (543) male/24% (170) female in 2012-2019 vs. 68% (140) male/32% (67) female in 2020-2021, p<0.001). There were relatively fewer women suffering hand trauma in the pre-COVID-19 cohort.

Cause of injury

**Figure 1:**
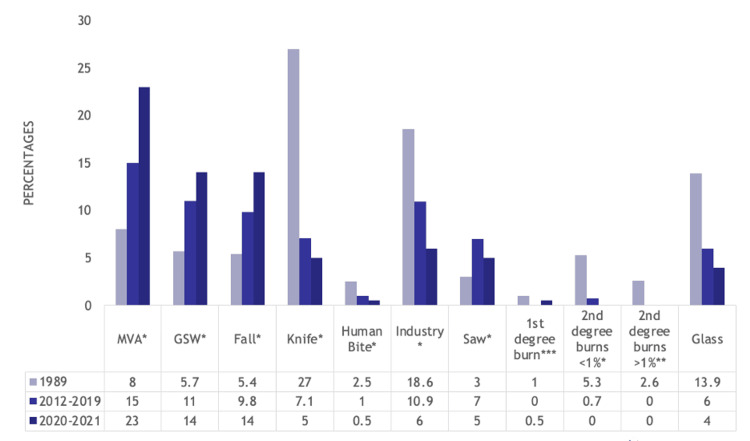
Bar graph showing statistically significant differences in cause of injury suffered by hand trauma patients between the 1989, 2012-2019, and 2020-2021 cohorts Numbers listed are percentages MVA: motor vehicle accident, GSW: gunshot wound *p<0.001, **p=0.025, ***p=0.023

In terms of cause of injury, the proportion of motor vehicle accidents (8% (78) vs. 15% (108) vs. 23% (47) p<0.001), gunshot wounds (5.7% (56) vs. 11% (77) vs. 14% (29), p<0.001), and falls (5.4% (53) vs. 9.8% (70) vs. 14% (29), p<0.001) increased over time from 1989 to 2012-2019 to 2020-2021. Knife injuries (27% (264) vs. 7.1% (51) vs. 5% (11), p<0.001), human bites (2.5% (24) vs. 1.0% (7) vs. 0.5% (1), p=0.025), industrial injuries (18.6% (182) vs. 10.9% (78) vs. 6% (12), p<0.001), second-degree burns <1% total body surface area (TBSA) (5.3% (52) vs. 0.60% (4) vs. 0% (0), p<0.001), second-degree burns >1% TBSA (2.6% (25) vs. 0% (0) vs. 0% (0), p<0.001), and glass injuries (13.9% (136) vs. 6.0% (43) vs. 4.0% (8), p<0.001) decreased over time. First-degree burn injuries were most common in the 1989 cohort (1.0% (10) vs. 0% (0) vs. 0.5% (1), p=0.023). Saw injuries were most common in the pre-COVID-19 cohort (3% (29) vs. 7.0% (50) vs. 5% (11), p<0.001). The other injuries were not statistically significant. The results of these trends are detailed in Figure 1.

Type of injury

**Figure 2:**
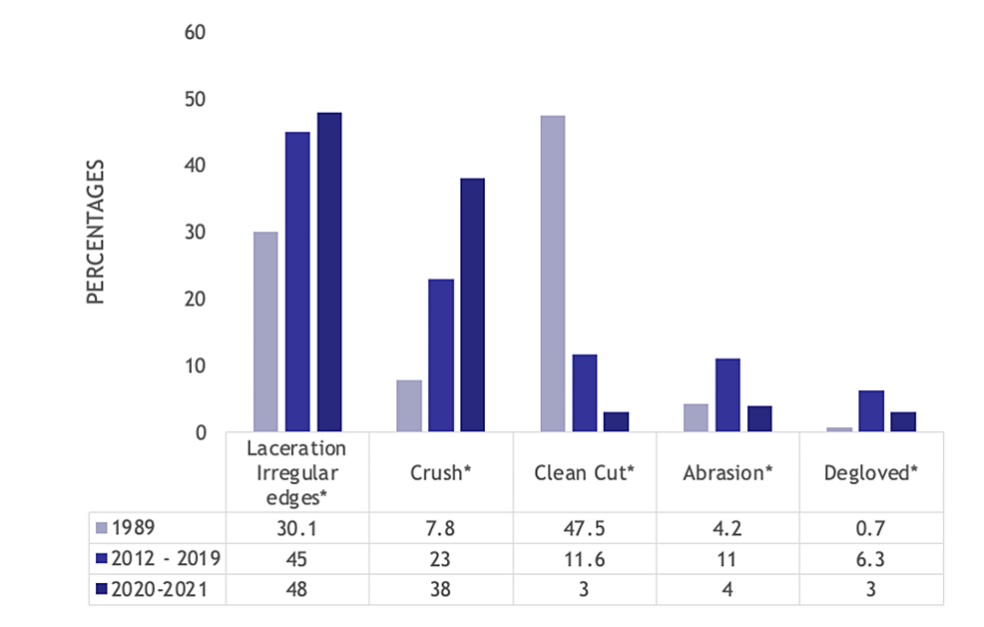
Bar graph showing statistically significant differences in type of injury suffered by hand trauma patients between the 1989, 2012-2019, and 2020-2021 cohorts Numbers listed are percentages *p<0.001

In terms of the type of injury, crush injuries (7.8% (76) vs. 23% (166) vs. 38% (78), p<0.001) and lacerations with irregular edges (30.1% (295) vs. 45% (324) vs. 48% (100), p<0.001) increased over time, whereas clean cut injuries decreased over time (47.5% (465) vs. 11.6% (83) vs. 3% (7), p<0.001). Abrasions (4.2% (41) vs. 11% (79) vs. 4% (9), p<0.001) and degloving injuries (0.7% (7) vs. 6.3% (45) vs. 3% (7), p<0.001) were most prevalent in the 2012-2019 cohort. These trends are detailed in Figure 2.

**Figure 3:**
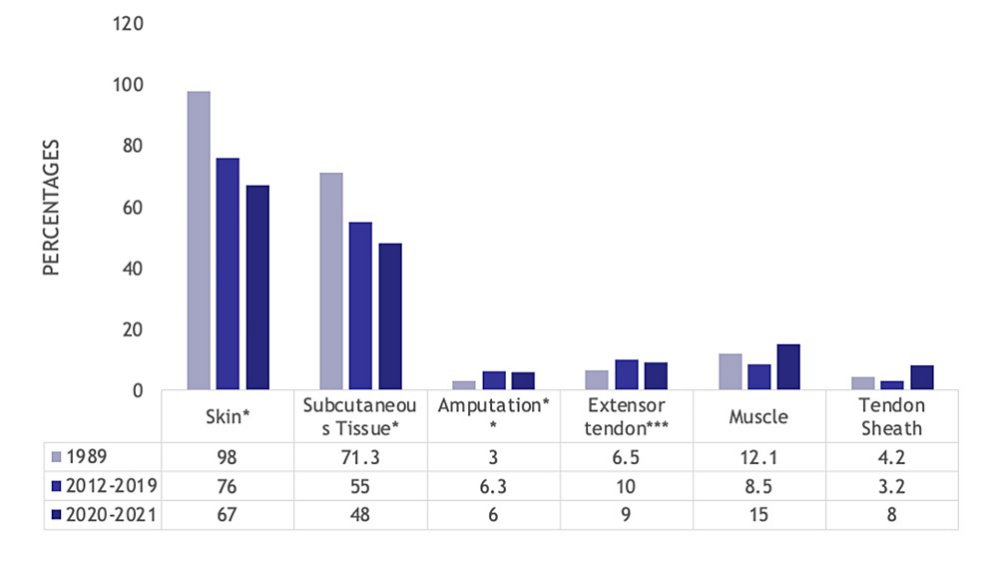
Bar graph showing statistically significant differences in types of soft tissue injury suffered by hand trauma patients between the 1989, 2012-2019, and 2020-2021 cohorts Numbers listed are percentages *p<0.001, **p=0.003, ***p=0.026, ****p=0.008, *****p=0.007

For soft tissue injuries, the number of skin (98% (959) vs. 74% (528) vs. 67% (139), p<0.001) and subcutaneous injuries (71.3% (698) vs. 57% (408) vs. 48% (100), p<0.001) decreased from 1989 to 2012-2019 to 2020-2021. Amputations (3% (29) vs. 6.3% (45) vs. 6% (12), p=0.003) and extensor tendon injuries (6.5% (64) vs. 10% (72) vs. 9% (19), p=0.026) were most common in the pre-COVID-19 period. Muscle injuries (12.1% (118) vs. 8.5% (61) vs. 15% (32), p=0.008) and tendon sheath injuries (4.2% (41) vs. 3.2% (23) vs. 8% (17), p=0.007) were most common during the COVID-19 era. These results are displayed in Figure 3.

**Figure 4:**
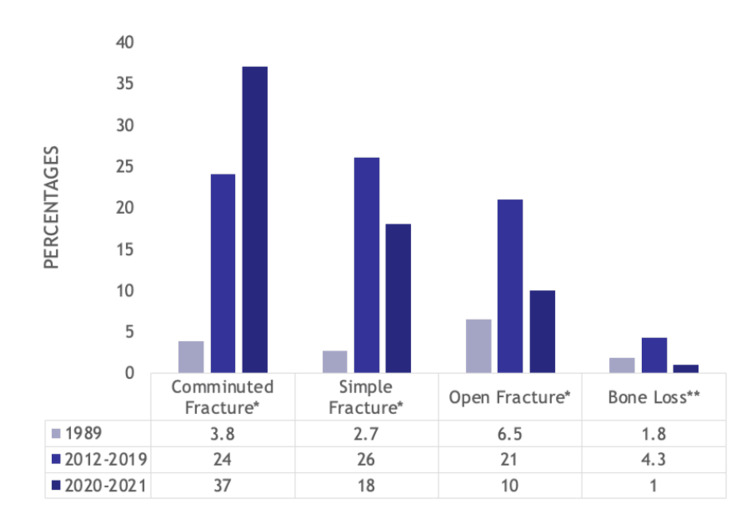
Bar graph showing statistically significant differences in types of bony injury suffered by hand trauma patients between the 1989, 2012-2019, and 2020-2021 cohorts Numbers listed are percentages *p<0.001, **p=0.002

For bony injuries, comminuted fractures (3.8% (37) vs. 24% (169) vs. 37% (77), p<0.001) became more prevalent from 1989 to 2012-2019 to 2020-2021, but simple fractures (2.7% (26) vs. 26% (188) vs. 18% (37), p<0.001), open fractures (6.5% (64) vs. 21% (149) vs. 10% (20), p<0.001) and bone loss (1.8% (18) vs. 4.3% (31) vs. 1% (2), p=0.002) were most prevalent in the pre-COVID-19 cohort. Regardless, bony fractures were generally more common in the 2010s/2020s and 2020s than in 1989. Such trends are displayed in Figure 4.

Treatments

**Figure 5:**
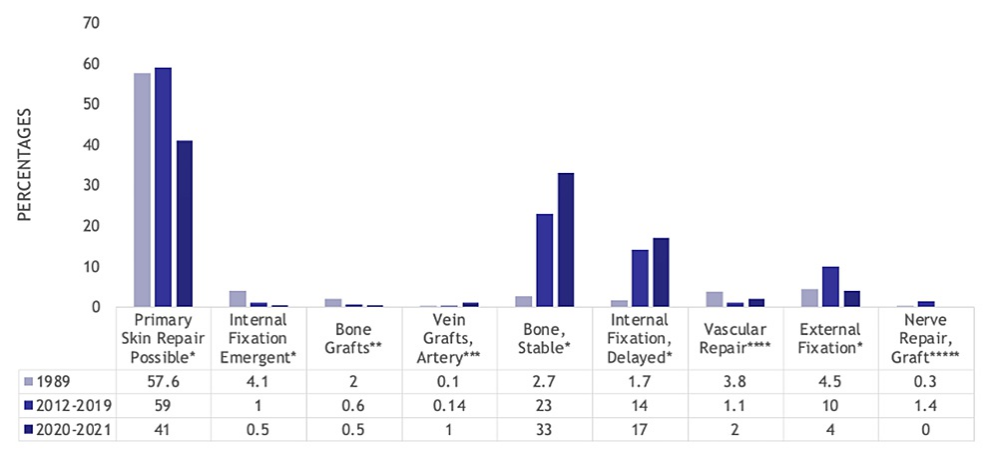
Bar graph showing statistically significant differences in treatments that hand trauma patients received between the 1989, 2012-2019, and 2020-2021 cohorts Numbers listed are percentages *p<0.001, **p=0.016, ***p=0.042, ****p=0.003, *****p=0.012

The treatments patients received differed between cohorts as well. From 1989 to 2012-2019 to 2020-2021, fewer patients could be treated with primary skin repair (57.6% (564) vs. 59% (419) vs. 41% (85), p<0.001), but fewer patients required emergent internal fixation (4.1% (40) vs. 1% (7) vs. 0.5% (1), p<0.001) or bone grafts (2% (20) vs. 0.6% (4) vs. 0.5% (1), p=0.016). More patients required vein grafts for arterial repair (0.1% (1) vs. 0.14% (1) vs. 1% (2), p=0.042), but more bony fractures could be treated non-operatively (2.7% (26) vs. 23% (166) vs. 33% (69), p<0.001) or with delayed internal fixation (1.7% (17) vs. 14% (101) vs. 17% (35), p<0.001). Vasculature repair was most common in the 1989 cohort (3.8% (37) vs. 1.1% (8) vs. 2% (5), p=0.003). The number of patients treated with external fixation (4.5% (44) vs. 10% (72) vs. 4% (8), p<0.001) and nerve repair with grafts (0.3% (3) vs. 1.4% (10) vs. 0% (0), p=0.012) were most prevalent in the pre-COVID-19 cohort. More patients required flaps (1.7% (17) vs. 084% (6) vs. 6% (13), p<0.001) in the COVID-19 era. These trends are displayed in Figure 5.

Hospitalizations, follow-up, and other trends

**Figure 6:**
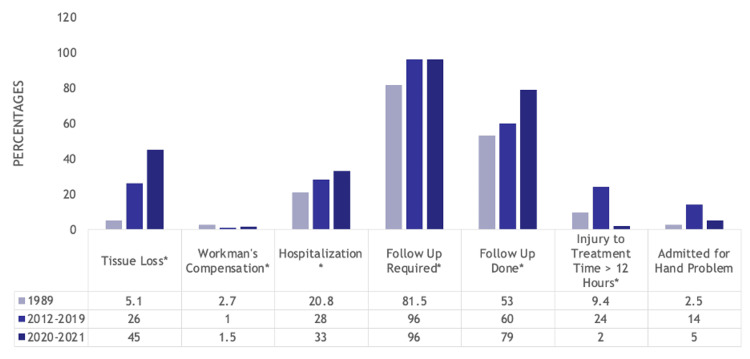
Bar graph showing statistically significant differences in terms of tissue loss, hospitalization, and referrals for hand trauma patients in the 1989, 2012-2019, and 2020-2021 cohorts Numbers listed are percentages *p<0.001

It is interesting to note factors such as tissue loss (5.1% (50) vs. 26% (186) vs. 45% (94), p<0.001) increased from 1989 to 2012-2019 to 2020-2021, whereas the proportion of workmen’s compensation cases decreased during the same time period (2.7% (26) vs. 1% (7) vs. 1.5% (3), p=0.039). More patients were hospitalized (20.8% (203) vs. 28% (198) vs. 33% (68), p<0.001), and more patients required follow-up (81.5% (798) vs. 96% (684) vs. 96% (198), p<0.001), but more follow-up was done (53% (519) vs. 60% (428) vs. 79% (156), p<0.001). Delay in treatment time (9.4% (92) vs. 24% (172) vs. 2% (5)) and being admitted for a hand problem (2.5% (24) vs. 14% (97) vs. 5% (11), p<0.001) were most common in the pre-COVID-19 cohort. Maximum recovery times were lower in 2020-2021 compared to 2012-2019 (p=0.042). These trends are noted in Figure 6.

Of note, there were no differences in complication percentages between cohorts. Each patient chart was reviewed in its entirety, from the date/encounter of the first consultation to the final follow-up date. Three patients (0.42%) were transferred out to other centers in the 2012-2019 cohort, whereas two patients (1.0%) were transferred out in the 2020-2021 cohort.

## Discussion

Currently, there are several studies reviewing hand trauma injuries that are limited by geography [3-7]. In a study done in Denmark and the Netherlands, it was found that hand injuries were most common in teenagers, mostly due to home or leisure accidents, mainly caused by objects and falls, mostly affecting fingers, and mostly causing open wounds, superficial injuries, and fractures, with a small proportion being admitted to the hospital [3]. There is some similarity to our study since our study did have a good deal of falls and superficial injuries and not many patients were hospitalized. Meanwhile, a Norwegian study commented on the incidence of moderate-severity open hand, wrist, or forearm injuries based on an abbreviated injury scale among males and females and the incidence of upper extremity amputations among males and females [4]. Another study done in Poland found that most hand injuries were in males and those under 30, 56.1% of patients had tendon injuries, 37.8% had skin loss, 24.1% had amputations, 9.6% had fractures, 6.1% had nerve damage, and 5.5% had joint damage [5]. Our study had a lower incidence of tendon or joint injuries or amputations but a higher incidence of fractures or nerve injuries. A study conducted in the UAE found that most injuries (67.1%) occurred in the workplace and that machines caused most injuries (36.2%), followed by heavy objects (20.5%) and falls (11%) [6]. Our incidence of falls was higher from 2020-2021, but lower in 1989 and from 2012-2019. Desai et al. found that hand trauma was most common in males (88%) and in the age group 25-35 years (36%) and most often due to machine injuries (52.5%), followed by traffic accidents (25%) and assaults (13.5%) [7]. While our study did not examine "machine injuries," it did examine industrial injuries, and our incidence of industrial injuries is lower than in other studies [6-7].

Several studies examined hand trauma during the COVID-19 lockdown [8-14]. Garude et al. found that there were more injuries from tools at home compared to the pre-COVID-19 cohort, which was also found by Ho et al., Paiva et al., Sugrue et al., and Fortane et al. [8-10,12-13]. Ho et al. also found that workplace and domestic violence injuries increased during the COVID-19 lockdown in Sydney, Australia [9]. In contrast, Fortane noted a decrease in workplace accidents and sports accidents but an increased severity of hand and upper limb trauma [13]. Our study did not look at home injuries vs. workplace injuries, but we did find that industrial injuries decreased after COVID-19, similar to Fortane et al. [13]. However, we found that sports accidents did not differ between our pre- and post-COVID-19 cohorts.

Meanwhile, Paiva et al. found that there was no difference in injury severity or need for operating room or emergency department interventions during the COVID-19 lockdown [10]. Atia et al. found there was a greater intervention rate during the COVID-19 lockdown but no differences in the types of cases [11]. Rajput et al. found that road traffic collisions decreased and bike-related injuries increased, which is in contrast to our findings of increased motor vehicle accidents but no differences in bike accidents [14].

Shah et al. and Lee et al. looked at the epidemiology of pediatric hand injuries; we did not examine pediatric injuries in our study [15-16]. Regardless, Shah et al. found that the number and rate of hand injuries decreased from 1990 to 2009, most injuries occurred in males, most injuries occurred at home, and most injuries were lacerations; Lee et al. found that fractures were the most common injury type and most injuries were due to falls [15-16].

Only one study looked at differences in hand trauma between two non-consecutive years in Sweden and found that mechanisms of injury differed between 1989 and 1997 (more falls and cuts in 1997, fewer crushes, burns, and traffic accidents), and the number of days of sick leave differed as well (fewer days in 1997) [2]. Some trends were the same in our study, such as an increase in falls and a decrease in burns. Their study, however, found that cuts increased over time, while we found that they decreased over time. Our study is the first retrospective review of trends in hand trauma injuries at a single institution in the United States over a 30-year time period and a global pandemic.

The mechanism of injury observed in our modern cohort revealed an increase in car accidents, gunshots, and fall-related hand injuries in comparison to the data from 1989. This trend can be attributed to the rise in personal vehicles, increased ease and access to firearms, and the aging of "baby boomers," resulting in frailty and falls. According to the United States Department of Transportation, there were 192,497 registered vehicles in 1994 (the earliest year related data was available) vs. 297,644 registered vehicles in 2020 [17]. Also, according to the CDC, there has been an increase in non-fatal injuries due to falls in 2020 (30%) vs. 2012 (28.3%). While non-fatal firearm injuries do not appear to have been nationally tracked by the CDC since 2015, in 2012, the earliest year for the pre-COVID-19 cohort of this study, the non-fatal injury percentage was 0.3% [18]. Fatal firearm injuries monitored by the CDC reveal that most data regarding firearm injuries in the U.S. is poorly reported with inconsistent tracking [19].

In contrast, the decrease in industrial injuries, glass injuries, and burn injuries may be due to improved workplace and household safety standards. According to the US Bureau of Labor Statistics, non-fatal occupational injury and illness incidence rates have decreased over time from 2003 to 2018 [20]. A decrease in knife injuries can be attributed to an increase in firearm use for interpersonal violence. However, the CDC statistics suggest otherwise, as the percentage of penetrating injuries in 2020 was 8.1% vs. 7.6% in 2012. The discrepancy may be due to local or regional factors such as the fact that firearms are easier to obtain or gun ownership is more common in certain states than others, hence why penetrating injuries increased nationally overall (it may be more difficult to obtain firearms in certain areas but not knives) but not in our cohort [21].

The increase in crush injuries and lacerations with irregular edges naturally corresponds with the increased incidence of high-energy causes of hand trauma, such as high-speed car accidents or gunshot injuries, whereas the decrease in clean-cut injuries would correspond with the decrease in knife trauma [18,22]. The increased prevalence of degloving injuries and abrasions in the pre-COVID-19 era may imply that hand trauma was more severe from 2012-2019, even though the incidences of falls/motor vehicle accidents/gunshot wounds were higher from 2020-2021. The devolving injuries and abrasions may also be less severe during COVID-19 since more patients were injured at home in lower-energy situations than at work, as found in several studies previously mentioned [8-10,12-13].

The number of patients with skin and subcutaneous injuries decreased from 1989 in comparison to our 2012-2021 population, while those suffering amputations, extensor tendon, muscle, or tendon sheath damage increased. The latter trend corresponds with causes of trauma resulting in higher energy injuries, although the former trend argues against it [23]. Interestingly, while comminuted fractures increased progressively through the three time periods, simple fractures, open fractures, and fractures with bone loss were most common in the pre-COVID-19 cohort. This trend may imply that the trauma suffered from 2012-2019 was generally more intense than that suffered from 2020-2021. Some studies looking at hand trauma during the pandemic found that there were fewer emergency department consultations but no differences in injury severity; another found that hand trauma severity increased but occupational and sports injuries decreased; a third one found that do-it-yourself/household injuries became more common, and those are generally lower energy and thus less severe [9-11]. The literature has variable findings.

In terms of treatment, fewer patients could be treated with primary skin repair during COVID-19. This corresponds with an increased incidence of high-energy trauma that results in skin injuries that cannot be treated with primary skin repair. More patients could be treated non-operatively for bony fractures or with delayed internal fixation in the more recent cohorts compared to 1989. However, it was found that external fixation was most common from 2012-2019, which is consistent with our data showing higher energy injuries in the pre-COVID-19 cohort [23]. The decreased incidence of primary vasculature repair and the increased use of grafts for nerve repairs in the pre-COVID-19 cohort imply more severe trauma for this time period, although the increased incidence of arterial repairs requiring vein grafts argues for more severe trauma from 2020-2021. Fewer patients required emergent internal fixation in the modern cohorts; this trend could imply an increased threshold for emergent operative treatment of fractures or a change in the nature of presenting injuries, such as an increased incidence of milder hand injuries not requiring emergent treatment.

Finally, it is noteworthy that, though more patients required follow-up, there was a positive correlation between patients who followed up as recommended, particularly in the post-COVID-19 era. Some studies have found that follow-up decreased during the pandemic; however, our results opposed this finding, and we postulate that the implementation of virtual work hours and flexibility of time during and after the global pandemic allowed for improved follow-up in our patients [24-25].

The percentage of hospitalized patients increased as well, either implying that there is a lower threshold to admit patients or that patients are suffering from more severe high-energy injuries, thus necessitating hospitalization. The number of patients suffering from tissue loss increased over time as well, which would correspond with higher energy mechanisms of trauma. However, the time to maximum recovery decreased, which can be attributed to more efficient and higher-quality care over the last several decades.

Study limitations involved the difficulty associated with prospective data collection in 1989 compared to retrospective data collection from 2012 to 2021. Therefore, while we compared data regarding hand injuries in 1989 only to hand injuries spanning from 2012 to 2021, our modern cohort data was not as robust. The retrospective nature of some of the data allows us to speculate causes for the observed trends but does not allow us to determine causation.

Our second limitation is regarding the size of the cohorts between 1989 and 2012-2021. There were many more patients in the 1989 cohort comprised of one year than in the 2012-2021 cohort. This discrepancy may be due to the fact that other hospitals, private clinics, and centers, such as Central Mississippi Medical Center (CMMC), opened in the latter years, leading to less hand trauma primarily presenting to the UMMC [26]. For example, CMMC admitted 5469 patients from July 2009 to June 2019 and noted an increase in nonburn hand cases from 92 in Year 1 to 1069 in Year 10, though not much other data is reported [26]. Furthermore, while the UMMC is the only level I trauma center in the state, it is not a burn center or a replantation center, so those patients generally do not present to UMMC. However, patients with burns that our team felt comfortable treating or patients with devascularized digits, hands, or forearms (with tissue still partially attached) requiring vascular repair were treated at UMMC.

Third, the retrospective data was collected via chart review of the electronic medical record, and charts were queried based on inpatient consult orders and search terms such as “hand,” “finger,” “digit,” or “wrist.” There may have been patients who suffered from hand trauma whom the emergency room treated without specialty consultations, or there may have been consults called without any consultation orders. Finally, some differences are statistically significant, but the absolute differences are small (such as differences in the incidence of human bites, burns, the need for vein grafts, the need for bone grafts, etc.); hence, these differences may not be clinically relevant.

## Conclusions

Our study is the first known study evaluating hand injuries in a modern setting in comparison to 1989 from a single, level I trauma center while studying the additional external factor of a global pandemic. Since the late 1980s, there has been a dramatic change in the number of personal vehicles, the accessibility of firearms, and the improvement of safety protocols with industrialization and technology. From our study, we can conclude that there is now more high-energy trauma to the hand from gunshots and car accidents compared to 1989 when there were more injuries from glass and knife wounds. This change resulted in significantly damaged skin and soft tissue, irregular wound edges, and increased hospitalizations in our patient population. Notably, patient follow-up was more commonly recommended, and surprisingly, patients had improved follow-up post-pandemic, which we hypothesize could be due to the flexibility of virtual work hours.

This data can help us focus our efforts when it comes to treating hand injuries, particularly those occurring in Mississippi or similar areas. Trainees can be made aware that more injuries will be due to motor vehicle accidents, gunshot wounds, and falls and should be trained on how to treat injuries resulting from these mechanisms. A greater emphasis can be placed on teaching trainees how to treat crush injuries, irregular lacerations, muscle injuries, tendon injuries, and comminuted fractures, and how to perform more delayed internal fixation or vessel repairs requiring vein grafts, since those are more common now. New innovations and treatments may also be directed toward these injuries.

Through this paper, we have an improved perspective on the nature and extent of hand injuries in modern times. This information can result in the initiation and refinement of management protocols for hand injuries and allow for improved outcomes. Additionally, by understanding the role of environmental factors (geography, occupation, access to firearms, availability of public transportation, and socioeconomic status) on hand injuries, which can cause severe functional impairment to a patient, more robust safety policies can be enacted. Our goal with this paper is to relay the changes to the mechanism of hand trauma over the last three decades. By adding to the growing data, we hope to implement efficient and optimal care for hand injuries and additionally improve patient safety and functionality.

## References

[1] Verdan C (2000). The history of hand surgery in Europe. J Hand Surg Br.

[2] Rosberg HE, Dahlin LB (2004). Epidemiology of hand injuries in a middle-sized city in southern Sweden: a retrospective comparison of 1989 and 1997. Scand J Plast Reconstr Surg Hand Surg.

[3] Larsen CF, Mulder S, Johansen AM, Stam C (2004). The epidemiology of hand injuries in The Netherlands and Denmark. Eur J Epidemiol.

[4] Atroshi I, Rosberg H (2001). Epidemiology of amputations and severe injuries of the hand. Hand Clin.

[5] Dębski T, Noszczyk BH (2021). Epidemiology of complex hand injuries treated in the plastic surgery department of a tertiary referral hospital in Warsaw. Eur J Trauma Emerg Surg.

[6] Grivna M, Eid HO, Abu-Zidan FM (2016). Epidemiology of isolated hand injuries in the United Arab Emirates. World J Orthop.

[7] Desai B, Makwana H, Shah D, Patel PR (2018). Epidemiology of hand injuries in adults presenting to a tertiary trauma care centre: a descriptive study. Int J Orthop Sci.

[8] Garude K, Natalwala I, Hughes B, West C, Bhat W (2020). Patterns of adult and paediatric hand trauma during the COVID-19 lockdown. J Plast Reconstr Aesthet Surg.

[9] Ho E, Riordan E, Nicklin S (2021). Hand injuries during COVID-19: lessons from lockdown. J Plast Reconstr Aesthet Surg.

[10] Paiva M, Rao V, Spake CS (2020). The impact of the COVID-19 pandemic on plastic surgery consultations in the emergency department. Plast Reconstr Surg Glob Open.

[11] Atia F, Pocnetz S, Selby A, Russell P, Bainbridge C, Johnson N (2020). The effect of the COVID-19 lockdown on hand trauma surgery utilization. Bone Jt Open.

[12] Sugrue CM, Sullivan P (2020). The effect of the ongoing COVID-19 nationwide lockdown on plastic surgery trauma caseload?. J Plast Reconstr Aesthet Surg.

[13] Fortané T, Bouyer M, Le Hanneur M (2021). Epidemiology of hand traumas during the COVID-19 confinement period. Injury.

[14] Rajput K, Sud A, Rees M, Rutka O (2021). Epidemiology of trauma presentations to a major trauma centre in the North West of England during the COVID-19 level 4 lockdown. Eur J Trauma Emerg Surg.

[15] Shah SS, Rochette LM, Smith GA (2012). Epidemiology of pediatric hand injuries presenting to United States emergency departments, 1990 to 2009. J Trauma Acute Care Surg.

[16] Lee A, Colen DL, Fox JP, Chang B, Lin IC (2021). Pediatric hand and upper extremity injuries presenting to emergency departments in the United States: epidemiology and health care-associated costs. Hand (N Y).

[17] (2022). New Vehicle Classification for 2020 and Later Years. https://www-fars.nhtsa.dot.gov/Main/index.aspx.

[18] (2022). Nonfatal Injury Data. https://www.cdc.gov/injury/wisqars/nonfatal.html.

[19] Kaufman EJ, Delgado MK (2022). The epidemiology of firearm injuries in the US: the need for comprehensive, real-time, actionable data. JAMA.

[20] (2022). Injuries, Illnesses, and Fatalities. https://www.bls.gov/iif/soii-charts-2018.pdf.

[21] (2024). Gun Ownership in America. https://www.rand.org/research/gun-policy/gun-ownership.html.

[22] Goodman AD, Got CJ, Weiss AC (2017). Crush injuries of the hand. J Hand Surg Am.

[23] Tintle SM, Baechler MF, Nanos GP 3rd, Forsberg JA, Potter BK (2010). Traumatic and trauma-related amputations: part II: upper extremity and future directions. J Bone Joint Surg Am.

[24] Abebe W, Worku A, Moges T (2021). Trends of follow-up clinic visits and admissions three-months before and during COVID-19 pandemic at Tikur Anbessa specialized hospital, Addis Ababa, Ethiopia: an interrupted time series analysis. BMC Health Serv Res.

[25] Spalletta G, Porcari DE, Banaj N, Ciullo V, Palmer K (2020). Effects of Covid-19 infection control measures on appointment cancelation in an Italian outpatient memory clinic. Front Psychiatry.

[26] Lineaweaver WC, Mullins RF (2021). Practice diversity and burn center growth: a 10-year profile of a state’s only burn center. Ann Plast Surg.

